# Advances in UAV Remote Sensing for Monitoring Crop Water and Nutrient Status: Modeling Methods, Influencing Factors, and Challenges

**DOI:** 10.3390/plants14162544

**Published:** 2025-08-15

**Authors:** Xiaofei Yang, Junying Chen, Xiaohan Lu, Hao Liu, Yanfu Liu, Xuqian Bai, Long Qian, Zhitao Zhang

**Affiliations:** 1 College of Water Resources and Architectural Engineering, Northwest A&F University, Xianyang 712100, China; xiaofei-yang@nwafu.edu.cn (X.Y.);; 2 Key Laboratory of Agricultural Soil and Water Engineering in Arid and Semiarid Areas, Ministry of Education, Northwest A&F University, Xianyang 712100, China; 3 Xinjiang Research Institute of Agriculture in Arid Areas, Northwest A&F University, Xianyang 712100, China

**Keywords:** UAV-based remote sensing, crop water status, crop nutrient status, precision agriculture

## Abstract

With the advancement of precision agriculture, Unmanned Aerial Vehicle (UAV)-based remote sensing has been increasingly employed for monitoring crop water and nutrient status due to its high flexibility, fine spatial resolution, and rapid data acquisition capabilities. This review systematically examines recent research progress and key technological pathways in UAV-based remote sensing for crop water and nutrient monitoring. It provides an in-depth analysis of UAV platforms, sensor configurations, and their suitability across diverse agricultural applications. The review also highlights critical data processing steps—including radiometric correction, image stitching, segmentation, and data fusion—and compares three major modeling approaches for parameter inversion: vegetation index-based, data-driven, and physically based methods. Representative application cases across various crops and spatiotemporal scales are summarized. Furthermore, the review explores factors affecting monitoring performance, such as crop growth stages, spatial resolution, illumination and meteorological conditions, and model generalization. Despite significant advancements, current limitations include insufficient sensor versatility, labor-intensive data processing chains, and limited model scalability. Finally, the review outlines future directions, including the integration of edge intelligence, hybrid physical–data modeling, and multi-source, three-dimensional collaborative sensing. This work aims to provide theoretical insights and technical support for advancing UAV-based remote sensing in precision agriculture.

## 1. Introduction

Crop water and nutrient status are critical determinants of agricultural productivity. Accurately characterizing their spatial and temporal distribution is essential for enhancing water use efficiency, optimizing fertilization practices, and ensuring food security [1,2]. Conventional monitoring of water and nutrient status has relied on manual sampling, point-based measurements, and empirical judgment. These methods are labor-intensive, lack temporal responsiveness, and offer limited spatial representativeness, rendering them inadequate for large-scale, high-frequency, and precision agricultural management. To improve the timeliness and accuracy of water and nutrient monitoring, remote sensing—characterized by its non-contact nature, wide spatial coverage, and operational efficiency—has become a critical tool for agricultural information acquisition.

Unmanned Aerial Vehicles (UAVs), with their flexible flight capabilities, operational simplicity, and cost-effectiveness, have become a key focus in agricultural remote sensing [3,4]. Compared with satellite- and manned aircraft-based systems, UAV-based remote sensing provides higher spatial resolution, greater temporal flexibility, and better repeatability, making it particularly well-suited for fine-scale agricultural monitoring at the field level [5]. Equipped with multispectral, hyperspectral, thermal infrared (TIR), and microwave sensors, UAVs can rapidly acquire multidimensional data, including canopy structure, spectral reflectance, and temperature distribution. This enables accurate diagnosis of crop water stress, nitrogen status, and optimal fertilization timing [6,7,8].

Advances in sensor technology, flight control systems, and data processing algorithms have driven substantial progress in UAV-based remote sensing for monitoring crop water and nutrient status. Methodological frameworks based on vegetation indices (VIs), thermal indicators, spectral inversion models, and multi-source data-driven algorithms have enabled high-precision estimation of crop parameters such as water status, nitrogen content, and canopy structure [9,10,11,12,13]. The quality and utility of UAV-based remote sensing data have been markedly enhanced by techniques such as image stitching, radiometric correction, segmentation, and data fusion [14].

Despite these advances, several challenges persist in scaling UAV-based remote sensing applications to large agricultural areas. Key challenges include the influence of crop growth stages and canopy coverage on spectral responses, scale mismatches and mixed pixel effects, environmental interference from illumination and meteorological conditions, and the complexity and low automation of current data processing workflows. In particular, the stability and adaptability of remote sensing models require further improvement to perform reliably across diverse crops, regions, and management conditions.

**Figure 1:**
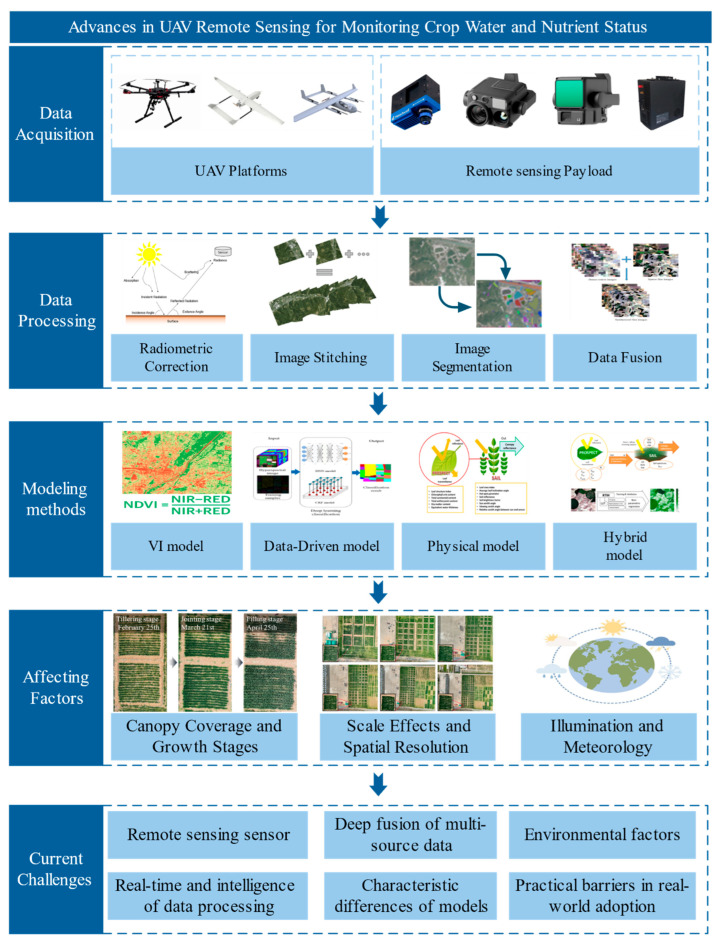
Framework of research progress on UAV-based remote sensing for monitoring crop water and nutrient status.

Based on this, the review aims to comprehensively examine the key technologies and research progress of UAV-based remote sensing in monitoring crop water and nutrient status. It systematically reviews the principles, advantages, and application scenarios of different monitoring methods, while analyzing the challenges and development trends in practical applications. To enhance the clarity of the technical workflow, a conceptual framework (Figure 1) is proposed, illustrating the end-to-end process of UAV-based remote sensing for crop monitoring—from sensor selection and data acquisition, through preprocessing and modeling, to field-level decision support. This review seeks to provide theoretical insights and technical references for the development of an efficient, intelligent, and scalable agricultural water and nutrient monitoring system.

## 2. Data Acquisition and Processing

High-quality data acquisition and standardized processing are fundamental to UAV-based remote sensing for monitoring crop water and nutrient status. UAV platforms equipped with various types of remote sensing sensors can capture multidimensional information reflecting crop physiological conditions and field environments. These data support parameter extraction, spatiotemporal variation analysis, and diagnostic model development for crop water and nutrient monitoring. However, raw remote sensing data are often affected by platform motion, atmospheric conditions, and illumination variability, leading to radiometric errors, geometric distortions, and noise contamination, which constrain their direct applicability. Therefore, preprocessing steps—such as radiometric correction, image stitching, image segmentation, and multi-source data fusion—are required to convert raw imagery into standardized data products suitable for quantitative analysis, thereby providing a reliable foundation for accurate and efficient monitoring of crop water and nutrient dynamics. This section outlines the main UAV platforms and remote sensing sensors currently used for monitoring crop water and nutrient status and introduces the key steps in remote sensing data processing.

### 2.1. UAV Platforms

In agricultural remote sensing, the choice of UAV platform directly affects the efficiency, cost, and suitability of data acquisition in different contexts. UAVs are commonly classified by rotor configuration into multirotor, fixed-wing, and hybrid-wing types [15]. The main technical parameters of these UAV platforms are summarized in Table 1, providing a quick comparison of endurance, altitude capability, cost, and maintenance requirements.

Multirotor UAVs typically have more than three rotors, with common configurations including quadcopters, hexacopters, and octocopters. Lift is generated by high-speed rotor rotation driven by electric motors, and flight attitude is controlled by adjusting individual rotor speeds. These UAVs offer advantages in vertical takeoff, landing, and stable hovering. However, their endurance is limited by battery capacity and power system constraints. They are primarily used for high-resolution data acquisition at the field scale.

Fixed-wing UAVs utilize an aerodynamic design similar to that of conventional aircraft, generating lift through airflow velocity differences above and below the wings. With their efficient aerodynamic structure, these UAVs offer advantages such as long endurance, high payload capacity, and extended flight range. However, they require longer runways for takeoff and landing or depend on catapult launches and gliding, which imposes greater demands on field conditions. They are mainly used for high-resolution data acquisition over irrigation districts and small watershed.

Hybrid-wing UAVs integrate the advantages of both multirotor and fixed-wing platforms. During takeoff and landing, they operate in multirotor mode to enable vertical takeoff and landing without requiring designated runways. During cruising, they switch to fixed-wing mode, utilizing aerodynamic lift for efficient long-distance flight. This design combines the flexibility of multirotor systems and the long endurance and range of fixed-wing platforms, enhancing operational efficiency and spatial coverage while maintaining adaptability in complex environments. However, their complex structure results in higher manufacturing costs and greater maintenance requirements. They are mainly used for applications requiring both vertical takeoff and long-range flights under complex field conditions.

### 2.2. Remote Sensing Payload

Remote sensing sensors are essential payloads for acquiring data on surface features. Based on their operating spectral bands, they are classified into optical, TIR, and microwave types, each providing complementary data for monitoring crop water and nutrient status. The key technical parameters and representative applications of these sensor types are summarized in Table 2, which provides a concise overview of their spectral ranges, typical payloads, and main agricultural monitoring uses.

Among optical sensors, multispectral and hyperspectral sensors are the most commonly used. Multispectral sensors detect reflected solar radiation in the visible and near-infrared (NIR) range (0.4–1.1 μm) and are widely applied in agriculture. Due to their relatively low cost and high spatial and temporal resolution, multispectral sensors are extensively used in agricultural water and nutrient monitoring, particularly for calculating various VIs to assess crop water stress status, nitrogen status, and leaf area index (LAI) [16,17,18]. Compared with multispectral sensors, hyperspectral sensors capture hundreds of contiguous narrow spectral bands spanning 0.4–2.5 μm range. Their high spectral resolution enables superior inversion accuracy in applications such as soil organic matter estimation, crop nutrient deficiency diagnosis, and crop water stress status [19,20,21].

TIR sensors detect longwave thermal radiation emitted from the land surface, typically in the 8–14 μm range, enabling retrieval of land surface temperature (LST). This capability makes them essential for detecting crop water stress and estimating evapotranspiration. Under water stress, reduced transpiration causes canopy temperature to rise. TIR sensors are sensitive to these thermal changes, enabling effective diagnosis of crop water stress [22,23]. While highly effective for water status monitoring, TIR data provide limited information for assessing crop nutrient status, as nutrient deficiencies generally do not induce direct and measurable changes in surface thermal emissions.

Microwave remote sensing plays an important role in crop water and nutrient monitoring due to its all-weather, day-and-night capability and sensitivity to the dielectric properties of surface materials. It is typically categorized into active and passive systems, each with distinct mechanisms and advantages.

Active microwave sensing, exemplified by Synthetic Aperture Radar (SAR), transmits microwave pulses and recording their backscattered signals to generate high-resolution information about the land surface. Analysis of SAR data with varying frequencies and polarization modes enables the retrieval of canopy water content (e.g., vegetation or leaf water content), which directly indicates crop water stress [24]. In addition, SAR’s sensitivity to surface roughness facilitates indirect estimation of soil moisture, since soil water content modulates the dielectric constant and alters surface scattering behavior.

Passive microwave sensing, typically using microwave radiometers, detects naturally emitted microwave radiation from the land surface, expressed as brightness temperature. Brightness temperature depends on the physical temperature and emissivity of the surface, which are influenced by soil moisture, vegetation water content, and canopy coverage. These parameters make passive microwave observations valuable for large-scale soil and vegetation moisture retrieval.

### 2.3. Data Processing

Raw remote sensing images acquired by UAVs capture digital signals influenced by surface features, atmospheric conditions, and sensor characteristics. To ensure accurate retrieval of crop physiological and biochemical parameters, raw data must be preprocessed to produce consistent and reliable reflectance products. The UAV remote sensing data processing workflow typically includes radiometric correction, image stitching, segmentation, and data fusion.

#### 2.3.1. Radiometric Correction

Radiometric correction is essential for quantitative remote sensing analysis. Its goal is to eliminate inconsistencies arising from solar irradiance variability, terrain effects, and sensor errors. Since the advent of satellite remote sensing, radiometric correction techniques for satellite imagery have been extensively developed and are now relatively mature [25,26,27,28]. In contrast, radiometric correction for UAV-based remote sensing imagery remains an emerging research area. Because of the low flight altitude of UAVs, atmospheric effects during image acquisition are significantly reduced compared with satellite-based systems. As a result, atmospheric correction procedures are typically simplified, focusing on solar irradiance variability, terrain-induced effects, and intrinsic sensor biases [29]. During repeated flights or extended UAV missions, acquired imagery is often affected by substantial variations in illumination conditions. Without correction, radiometric discrepancies between images can reduce the stability of inversion results and limit model generalizability.

Multispectral systems, owing to their simple structure, low cost, and broad applicability, are the most widely used imaging systems in UAV-based remote sensing. To address radiometric inconsistencies, Luo et al. [30] proposed a Piecewise Empirical Linear (PEL) method for estimating reflectance across diverse surface types, which significantly improved correlations between remote sensing indices and crop parameters. Using the MicaSense Altum system as a case study, Wang et al. [31] compared seven radiometric correction methods and found that relying on a single reference panel captured at one time point yielded poor results under rapidly changing illumination conditions. To improve reflectance consistency, they proposed using multiple reference panel placements combined with flight altitude calibration. In addition, optimizing low-cost reference panel materials has also garnered research attention. Rosas et al. [32] evaluated the reflectance performance and durability of various materials under different climatic conditions and recommended matte-coated wood panels and synthetic leather as practical alternatives, providing physical support for radiometric correction in low- to mid-cost UAV systems.

TIR cameras are widely used for detecting crop water stress and estimating evapotranspiration; however, their uncooled design makes them vulnerable to thermal drift and ambient temperature fluctuations, often leading to brightness temperature shifts and vignetting effects. To address this issue, Aragon et al. [33] developed a calibration function based on blackbody targets and ambient temperature responses, applicable to various infrared camera models (e.g., FLIR, TeAx), which significantly improves temperature estimation accuracy. Elfarkh et al. [34] found that flight altitude, acquisition time, and meteorological conditions significantly affect the consistency of LST. They recommended acquiring TIR data during radiometrically stable periods to ensure higher data quality. Wang et al. [35] further proposed the DRAT method, which integrates probability density fitting with a radiative transfer model to eliminate temporal drift errors without relying on extensive ground-based observations.

Although hyperspectral imaging systems provide high spectral resolution and fine classification capability, they are highly sensitive to platform orientation and illumination changes, often resulting in radiometric striping and geometric distortion. Jakob et al. [36] developed the MEPHySTo toolbox, which integrates geometric and radiometric processing of hyperspectral imagery and is suitable for high-precision applications such as mineral exploration. Building on this, Song et al. [37] proposed an integrated correction framework for such systems, combining attitude correction, solar irradiance modeling, geometric registration, and image stitching. This approach significantly enhances radiometric consistency and image continuity under large-scale operational conditions.

Collectively, these studies highlight the critical role of radiometric correction in ensuring the accuracy and consistency of UAV-based remote sensing data. As UAV applications expand in agricultural monitoring, developing robust, efficient, and scalable radiometric correction methods—particularly those that reduce reliance on physical targets and support multi-sensor integration—will be essential for reliable crop parameter retrieval across diverse spatial and temporal conditions.

#### 2.3.2. Image Stitching

UAV-based remote sensing has received considerable attention for its capability to capture high-spatial-resolution imagery. However, such high-resolution images typically cover only a limited portion of the land surface per frame. To address this limitation and acquire a comprehensive view of the study area, image stitching has become an essential technique. Image stitching combines multiple overlapping images into a larger composite, thereby improving the applicability of UAV-based remote sensing across diverse research and monitoring scenarios.

To address the dual challenge of achieving high spatial resolution and broad coverage in UAV imagery, researchers have proposed various image stitching techniques. Traditional image stitching techniques primarily rely on feature point matching strategies, such as the Scale-Invariant Feature Transform (SIFT) algorithm [38]. By extracting scale-invariant features and applying dynamic thresholding in the Difference-of-Gaussian (DoG) space, SIFT improves stitching efficiency and matching robustness in agricultural remote sensing. Ren et al. [39] developed a coarse-to-fine image stitching strategy based on a multilayer perceptron (MLP) to address spectral discrepancies and spatial misalignments in multispectral imagery. By incorporating feature-based error regression correction, this approach balances computational efficiency and stitching accuracy. Yang et al. [40] further proposed an enhancement-based approach to improve multispectral image stitching in complex riparian environments by reinforcing the spectral features of individual bands.

To mitigate common issues such as artifacts and structural distortions in traditional image stitching, Xu et al. [41] introduced a method that integrates Accelerated-KAZE (AKAZE) feature extraction with the As-Projective-As-Possible (APAP) model. By incorporating structure-preserving mechanisms and multi-level constraints, this approach improves structural consistency and visual coherence in stitched imagery. For high-dimensional data such as hyperspectral imagery, Mo et al. [42] proposed an image stitching method that integrates deep feature representations with a graph neural network (GNN). By incorporating spectral consistency correction and multi-scale fusion, their method achieves seamless, low-distortion, high-quality stitching results.

To address the challenges of multi-sensor and multi-modal image co-registration and stitching, Turner et al. [43] achieved spatial alignment and joint stitching of visible, multispectral, and TIR imagery in a study on Antarctic moss monitoring, with a root mean square error (RMSE) as low as 1.78 pixels. In addition, Kapil et al. [44] addressed low-contrast TIR image stitching by developing a texture mapping framework that uses synchronized RGB imagery as an intermediary. This approach improved the geometric consistency and radiometric quality of TIR orthomosaics.

#### 2.3.3. Image Segmentation

During early growth stages or under conditions of low planting density, high-resolution UAV imagery often exhibits mixed spectral responses due to insufficient vegetation cover. In such cases, image pixels can generally be classified into three categories: bare soil, crop canopy, and other background noise. If raw UAV imagery is directly used for vegetation monitoring or parameter inversion, background noise may reduce the descriptive capacity of remote sensing data and compromises model accuracy. Therefore, to improve the efficiency and accuracy of remote sensing data analysis, image segmentation is essential for isolating meaningful land surface components from raw imagery. In agricultural remote sensing, commonly used segmentation methods include threshold-based techniques, machine learning (ML) algorithms, and increasingly adopted deep learning (DL) approaches.

Traditional threshold-based segmentation methods, such as NDVI-based thresholding, typically require a manually defined fixed threshold to separate vegetated from non-vegetated regions [45]. Although computationally efficient and relatively easy to implement, the manual selection of threshold values is subjective and poorly adapted to varying illumination and land surface conditions across different images. To improve objectivity and adaptability, adaptive thresholding techniques such as the Otsu algorithm have been widely adopted. The Otsu algorithm automatically determines the optimal threshold by maximizing the between-class variance of two groups—foreground (e.g., vegetation) and background (e.g., soil)—from the image histogram, eliminating the need for manual intervention [46]. This approach enhances the robustness of threshold-based segmentation to some extent. However, whether using fixed or adaptive methods, segmentation performance often declines when significant spectral overlap among land surface components exists or when imagery is affected by noise or uneven illumination.

Supervised segmentation methods such as Support Vector Machines (SVM) and Random Forests (RF) rely on training samples to learn the spectral characteristics of land surface types [47]. These methods can achieve more accurate segmentation in complex scenarios, improving the ability to identify various land surface features. In recent years, DL approaches—particularly Convolutional Neural Networks (CNNs)—have demonstrated significant advantages in remote sensing image segmentation due to their strong capabilities in hierarchical feature extraction and spatial context modeling [48]. When processing farmland images with complex textured backgrounds and small-scale targets (e.g., individual seedlings), DL models can adaptively learn high-level semantic features, improving segmentation accuracy and model robustness.

Effective image segmentation removes bare soil, shadows, and other background interferences, improving the accuracy of vegetation information extraction and providing a more reliable data foundation for subsequent tasks such as crop parameter estimation, water stress detection, and field condition assessment. Therefore, for high-resolution UAV imagery, segmentation is an indispensable step in the preprocessing pipeline and plays a crucial role in improving the accuracy and stability of farmland remote sensing analysis.

#### 2.3.4. Data Fusion

In complex agricultural environments, a single sensor is often insufficient to capture the multidimensional growth characteristics of crops, including canopy structure, physiological and biochemical status, and water and thermal conditions. As a result, multi-sensor collaborative observation has become a dominant trend. Against this backdrop, data fusion techniques have been developed to integrate imagery from different sensors at spatial, spectral, temporal, and semantic level, aiming to enhance the accuracy and robustness of crop parameter retrieval.

Image fusion methods can be classified into three categories based on processing level: pixel-level, feature-level, and decision-level fusion. Pixel-level fusion integrates information directly at the pixel level and is suitable for data sources with similar spatial resolutions. For example, in cotton aphid monitoring, the Gram–Schmidt transformation was used to fuse multispectral and panchromatic images, enhancing the sensitivity of VIs to aphid infestation severity (R^2^ = 0.88) [49]. In rice straw burning detection, fusing infrared and visible imagery improved object detection performance, with mAP@0.5 increasing by approximately 5% [50]. Feature-level fusion combines intermediate features such as texture, spectral indices, and VIs for joint modeling. For instance, in cotton yield estimation, combining LiDAR-derived plant height and multispectral-based leaf chlorophyll content (LCC) in an XGBoost model improved prediction accuracy (R^2^ = 0.802) [51]. Similarly, in potato aboveground biomass estimation, a RF model combining texture, spectral, and meteorological features achieved the best performance (R^2^ = 0.79) [52]. Decision-level fusion integrates outputs from multiple models or sensors and is particularly suitable for handling heterogeneous data sources. In multimodal fusion frameworks, optical and SAR imagery can be modeled independently and then combined through weighted integration to improve the robustness of LAI and canopy cover estimation [53].

In recent years, multimodal fusion combined with DL modeling has become an important advancement in remote sensing data integration. Multimodal approaches typically integrate RGB, TIR, and multispectral imagery to improve the capacity of models to capture crop physiological traits and environmental conditions. For example, in a citrus orchard under drip irrigation, combining RGB, TIR, and multispectral data with a CNN-LSTM model improved the prediction accuracy of 5 cm soil moisture content (SMC), achieving R^2^ = 0.88 [54]. Song et al. [55] integrated visible imagery with enhanced multispectral data to generate outputs with higher spatial resolution and richer spectral information, improving the accuracy of sunflower lodging detection from 84.4% to 89.8%.

## 3. UAV-Based Remote Sensing Modeling Methods for Crop Water and Nutrient Status

### 3.1. VI-Based Approaches

In remote sensing-based crop monitoring, physiological parameters such as leaf water content and chlorophyll concentration are key indicators of water and nutrient status, but are often difficult to measure directly. VIs, as commonly used and effective remote sensing parameters, provide an indirect means of assessment by enhancing vegetation signals and suppressing non-target interference, such as soil background and illumination variability. This is typically achieved through band-based operations such as ratios, differences, or normalization. The theoretical foundation of VIs lies in the distinct spectral reflectance characteristics of vegetation across different wavelengths—particularly the strong contrast between visible and NIR bands—which are highly sensitive to variations in canopy structure, physiological activity, and water stress. Therefore, VIs play a vital role in the remote sensing-based inversion and assessment of crop water and nutrient status. Commonly used VIs are summarized in Table 3. These indices demonstrate strong adaptability across sensor types and spatial scales, serving as effective indicators of crop water and nutrient status.

#### 3.1.1. Applications of VIs in Monitoring Crop Water Status

VIs reflect canopy coverage and leaf water status and are therefore widely used to monitor soil moisture and crop water stress [69,70]. Drought stress reduces leaf water content and transpiration, altering spectral reflectance characteristics and leads to a decline in VI values. NDVI is widely used to monitor water stress; however, its sensitivity to soil background and illumination conditions limits its accuracy.

To improve monitoring accuracy, researchers have developed various water indices based on NIR and shortwave infrared (SWIR) bands. These indices are constructed by leveraging the distinct absorption and reflectance characteristics of vegetation at specific wavelengths (e.g., 970 nm, 1200 nm, 1450 nm, 1940 nm, and 2500 nm), which are closely associated with crop water content. Gao [71] proposed the Normalized Difference Water Index (NDWI), which estimates leaf water content using the reflectance difference between the 860 and 1240 nm bands. Building on this, Wang and Qu [72] introduced the Normalized Multiband Drought Index (NMDI), which integrates two strong water absorption bands at 1640 and 2130 nm. This index improves the detection of compound drought conditions and demonstrates greater stability in heterogeneous farmland. In addition to traditional VIs, recent studies have developed novel indices and their lightweight applications in orchards or crop-specific scenarios. For example, Gao et al. [73] developed two convolution-based VIs (CNRVI and CNGVI) and proposed a lightweight CVCR-Net model for root-zone soil moisture inversion in kiwifruit orchards. The model reduced both size and parameter count, enhancing deployment efficiency while maintaining accuracy, offering a novel approach to crop water monitoring on embedded platforms.

TIR remote sensing enables the analysis of the relationship between canopy temperature (Tc) and land surface evapotranspiration, focusing on how crop water stress influences canopy thermal radiation. In the 1960s, Tanner [74] was the first to employ the temperature difference between canopy and air (Tc–Ta) to qualitatively assess crop water status. Building on this concept, Idso et al. [75] introduced a non-transpiring baseline and proposed the prototype of the Crop Water Stress Index (CWSI), which incorporates both Tc–Ta and vapor pressure deficit (VPD). Later, Jackson et al. [76] integrated land surface energy balance theory into the model, establishing a physically based version of CWSI. This advancement enabled CWSI to be widely applied across crops, regions, and temporal scales as a robust indicator of crop water status. Compared with traditional VIs, CWSI exhibits greater stability in areas with low vegetation cover and can indirectly reflect root-zone moisture dynamics [77]. Owing to these features, CWSI serves as a reliable indicator for irrigation scheduling and water stress monitoring in precision agriculture.

In addition to vegetation- and temperature-based indices, recent studies have developed feature space analysis methods to integrate LST and VIs to improve soil moisture retrieval accuracy. Based on the surface energy balance principle, these methods construct a negatively correlated Ts–VI feature space, reflecting the mechanism whereby surface temperature rises under dry conditions and decreases in moist areas. Sandholt et al. [78] proposed the Temperature Vegetation Dryness Index (TVDI), which enables quantitative monitoring of regional-scale water stress by normalizing LST along the dry and wet edges of the Ts–VI feature space. However, the accuracy of the Ts–VI method is affected by atmospheric conditions, surface heterogeneity, and seasonal variations, necessitating cross-validation with ground-based measurements and multi-source data in practical applications.

Complementing optical and TIR methods, SAR technology offers advantages for soil moisture retrieval, especially under cloudy or low-light conditions. Zhang et al. [79] proposed a dual-polarization SAR vegetation index (DRVI), that integrates backscatter contrast and polarization parameters. Combined with the water-cloud model, this approach achieved accurate moisture estimation across crop types, significantly outperforming conventional indices such as NDVI and LAI.

In summary, remote sensing technologies based on VIs—ranging from optical water indices to canopy temperature and SAR polarization indices—have established a multidimensional, cross-scale framework for crop water diagnosis. This framework provides a theoretical basis and technical support for precision irrigation, water resource management, and early drought warning.

#### 3.1.2. Applications of VIs in Monitoring Crop Nutrient Status

Among the three primary macronutrients in crops, nitrogen has been the most extensively studied using remote sensing, with a relatively mature methodological framework. This is largely attributed to the pivotal role nitrogen plays in photosynthesis, where variations in N content affect chlorophyll synthesis and vegetation spectral reflectance—particularly the contrast between the visible and NIR bands. Since the 1980s, numerous nitrogen-sensitive VIs have been developed, providing a foundation for high-resolution spatial and temporal monitoring of crop nitrogen status [80].

NDVI, one of the earliest and most widely used indices, can detect nitrogen-induced variations. However, it often suffers from spectral saturation under dense canopy or high LAI conditions, limiting its effectiveness in monitoring crop nitrogen status during the mid to late growth stages [81]. To address this limitation, researchers have developed improved VIs by incorporating more sensitive spectral bands, such as the GNDVI [60], the MERIS Terrestrial Chlorophyll Index (MTCI) [82], and the Chlorophyll Index Red-Edge (CIred-edge) [83]. Notably, the inclusion of the red-edge spectral region (700–750 nm) has improved the sensitivity of these indices to nitrogen variation, making them effective for monitoring nitrogen status during the mid to late growth stages [84,85]. For example, Zhang et al. [86] found that the red-edge wavelength at 718 nm exhibited the highest correlation with leaf nitrogen concentration (LNC) (r = 0.92) when analyzing hyperspectral imagery of maize leaves under varying nitrogen treatments. Based on this band, the derived Normalized Difference Spectral Index (NDSI) and Ratio Spectral Index (RSI) achieved strong predictive performance, with R^2^ values of 0.90 and 0.86, respectively. Furthermore, nitrogen level classification based on these indices achieved an accuracy of 91.7%, underscoring the advantages of red-edge and NIR band combinations in nitrogen discrimination.

Progress has also been made in the remote sensing of other essential nutrients, such as phosphorus (P) and potassium (K), but related research remains at an early stage. The primary challenge in monitoring phosphorus is its low concentration and comparatively weak influence on spectral reflectance relative to nitrogen. Nevertheless, Wang et al. [87] utilized the successive projections algorithm (SPA) to identify key wavelengths from hyperspectral data and developed a three-band VI, which improved the estimation accuracy of phosphorus content in sedge leaves to R^2^ > 0.68, outperforming traditional two-band indices. Furthermore, the study emphasized that NIR and SWIR bands are particularly sensitive to phosphorus variation, indicating a promising direction for future large-scale phosphorus monitoring.

Potassium (K) plays an important role in regulating cellular osmotic balance, enzyme activity, and stress resistance, and displays distinct spectral response features in the red-edge and SWIR regions. Lu et al. [88] developed two novel SWIR indices—NDSI (R1705, R1385) and RSI (R1385, R1705)—to estimate potassium content in rice leaves, achieving R^2^ = 0.68. Incorporating the red-edge band to form a three-band combination, the predictive performance improved to R^2^ = 0.74. Similarly, Yang et al. [89] identified reflectance near 1883 and 2305 nm as strongly indicative of leaf potassium content in wheat. The partial least squares regression (PLSR) models produced R^2^ values of 0.74 and 0.65, respectively. Collectively, these findings highlight the important role of SWIR region in remotely sensing crop potassium status.

### 3.2. Data-Driven Approaches

With the increasing availability of high-dimensional, multi-source UAV-based remote sensing data, data-driven models have become essential for retrieving crop water and nutrient status. Traditional linear models, such as multiple linear regression (MLR) and PLSR have been widely used to establish quantitative relationships between VIs, spectral and thermal features, and crop physiological parameters [90]. However, the nonlinear and heterogeneous nature of remote sensing data often limits their accuracy and generalizability. In contrast, ML and DL methods offer powerful capabilities in nonlinear modeling, feature extraction, and temporal dynamics analysis. Recent advances in tree-based models [91], kernel methods [92], artificial neural networks (ANNs) [93], and deep architectures have significantly improved the performance of remote sensing-based crop status assessment [94]. The following sections review representative models within these categories.

#### 3.2.1. Data-Driven Approaches for Crop Water Status Estimation

Estimating crop water status from remote sensing data often presents challenges such as high dimensionality, strong nonlinearity, and multi-source data integration. Traditional parametric models struggle to capture the complex interactions between remote sensing features and crop water status, making data-driven approaches increasingly important. Early studies often employed linear regression models to quantify the relationships between remote sensing indicators—such as VIs (e.g., NDVI, NDWI) and LST—and crop water content. Zhang et al. [95] developed a leaf water content inversion model using ground-based hyperspectral data and evaluated several regression techniques. Results showed that PLSR consistently achieved the highest prediction accuracy across growth stages. However, linear models are limited in capturing nonlinear interactions between crops and environmental factors, which reduces both accuracy and generalizability.

To address the limitations of linear models, researchers have increasingly adopted ML approaches. Algorithms such as RF, SVM, and XGBoost have been successfully applied to remote sensing-based estimation of soil moisture and canopy water content, owing to their robust feature modeling capabilities and resistance to noise. For example, Chen et al. [96] developed the Thermal-Derived Drought Index (TDDI) using UAV-based multisource imagery and temperature features. By integrating this index with ML models such as RF and SVM, they estimated the water content of maize and sorghum. Results showed that RF achieved the highest prediction accuracy under both single-crop and multi-crop scenarios. Savchik et al. [97] employed RF and ANN models, integrating canopy spectral data and soil information to predict stem water potential in almond orchards. Divya Dharshini et al. [98] evaluated four ML algorithms for predicting relative water content under varying irrigation regimes using sorghum as a case study. SVM achieved the highest accuracy under irrigated conditions (R^2^ = 0.94), followed by XGBoost. Under rainfed conditions, XGBoost demonstrated greater robustness, while PLSR performed poorly in both scenarios.

With the increasing availability of large-scale and time-series remote sensing data, DL methods have demonstrated significant advantages in modeling dynamic changes in crop water status. Babaeian et al. [99] integrated UAV-based multispectral imagery with soil physicochemical properties to develop an AutoML framework for estimating root-zone soil moisture, achieving high accuracy (RMSE < 0.02 cm^3^/cm^3^). Yang et al. [100] proposed a UAV-based strategy for crop water status estimation by constructing a deep fusion framework with a dynamic model updating mechanism, which improving adaptability across growth stages. In addition, Yang et al. [101] applied continuous wavelet transform to extract features from hyperspectral and TIR data, integrating them into a neural network model to estimate leaf water content in winter wheat, achieving high accuracy (R^2^ > 0.9). These findings underscore the potential of integrating remote sensing feature fusion with DL for accurate crop water assessment.

#### 3.2.2. Data-Driven Approaches for Crop Nutrient Estimation

Crop nutrient content, particularly nitrogen (N), phosphorus (P), and potassium (K), is a key determinant of plant growth and yield. In recent years, remote sensing technologies—particularly hyperspectral and UAV-based platforms—have become important tools for assessing crop nutrient status due to their rapid, non-destructive, and large-scale monitoring capabilities. However, complex and nonlinear relationships between nutrient levels and remote sensing features often reduce the performance of traditional statistical models. In this context, data-driven approaches present a promising alternative for improving the accuracy and robustness of nutrient estimation.

Among ML methods, ensemble algorithms such as RF and XGBoost have been widely applied in nutrient estimation due to their strong feature selection and nonlinear modeling capabilities. Jiang et al. [102] achieved accurate estimation of leaf nitrogen content in *Annona squamosa* by integrating UAV-based hyperspectral data with ensemble learning models. Similarly, Zha et al. [103] combined UAV data with RF to improve prediction accuracy of the nitrogen nutrition index in rice (R^2^ = 0.94–0.96), outperforming both VIs (R^2^ = 0.43–0.63) and MLR (R^2^ = 0.54–0.75). These studies demonstrate the effectiveness of ensemble learning in capturing complex relationships between VIs and nutrient status.

With increasing dimensionality and spatiotemporal resolution of remote sensing data, DL approaches have shown clear advantages in nutrient monitoring. Chen et al. [104] developed a DL framework integrating hyperspectral reflectance and phenological information to predict nitrogen content in apple leaves, achieving R^2^ = 0.79 on the validation set. Xiao et al. [105] proposed a CNN model based on visible and NIR spectra, which predicted nitrogen concentration in cotton leaves (RMSE = 3.36) and classified nitrogen status with 83.34% accuracy. Zhang et al. [106] introduced a self-supervised spectral–spatial vision transformer (SSVT) that jointly learns spatial and spectral features, improving nitrogen status prediction in wheat (accuracy = 0.96).

Recent research highlights multi-source data fusion and cross-scale modeling as key directions in nutrient monitoring. Du et al. [107] developed an incremental learning model to predict LCC and LAI across crops including soybean, rapeseed, and wheat, achieving R^2^ values from 0.56 to 0.82. Similarly, Dehghan-Shoar et al. [108] constructed a physics-informed neural network that incorporates prior knowledge from radiative transfer models, improving the robustness of nitrogen estimation across scales (R^2^ = 0.71). These approaches offer valuable technical support for precision fertilization in agriculture.

### 3.3. Physically Based Approaches

Physically based models are the most mechanistically grounded approach in agricultural remote sensing, as they quantitatively simulate energy and matter transfer within crop systems to elucidate interactions between physiological processes and environmental conditions. Compared with empirical models, physically based models offer better interpretability and extrapolation capabilities, as they do not depend on region-specific training datasets. These advantages make them effective for large-scale crop water and nutrient monitoring across diverse spatial and temporal contexts. Based on their underlying mechanisms, these models can be categorized into three types: canopy radiative transfer models, microwave radiative transfer models, and energy balance models.

#### 3.3.1. Canopy Radiative Transfer Models

Canopy radiative transfer models constitute the theoretical basis for retrieving crop structural and physiological–biochemical parameters from remote sensing data. These models simulate the interactions of solar radiation with crop canopies, including absorption, reflection, transmission, and multiple scattering. Through such physical modeling, critical parameters—such as LAI, chlorophyll content, and leaf water content—can be quantitatively estimated from spectral reflectance, thereby providing support for precision water and nutrient management in agriculture.

Based on the canopy structural characteristics, radiative transfer models can be broadly classified into two categories: continuous medium models and discrete medium models. Continuous models assume a homogeneous canopy structure, where leaves are uniformly distributed along the vertical profile and spatial variability among individual plants is ignored. This approach is well-suited for crop canopies with dense coverage and uniform architecture. A representative example is the SAIL (Scattering by Arbitrarily Inclined Leaves) model, which uses a four-stream radiative transfer formulation to simulate the vertical propagation and scattering of solar radiation within the canopy [109]. The spectral characteristics of individual leaves are described using the PROSPECT model, which incorporates leaf internal structure, chlorophyll concentration, water content, and dry matter effects on reflectance and transmittance [110]. The combination of SAIL and PROSPECT forms the PROSAIL model, which is widely recognized as one of the most prevalent canopy radiative transfer models. With a limited number of input variables (e.g., LAI, Cab, Cw, and structure parameter N), the PROSAIL model can generate canopy reflectance spectra, making it a standard tool in remote sensing inversion studies [111].

In contrast, discrete models do not assume a homogeneous canopy structure. Instead, they account for the spatial distribution of individual plants and are typically based on geometric-optical theory. A representative example is the Li–Strahler geometric-optical model, which characterizes projection, occlusion, shadowing, and multiple scattering among plant components (e.g., individual crowns or crop rows) [112]. This model is suited for sparsely vegetated fields, agricultural plots with distinct row structures, and heterogeneous landscapes. Discrete models resolve the combined effects of direct illumination, canopy shadows, and background elements such as bare soil, making them advantageous for analyzing high-resolution UAV remote sensing imagery. Additionally, these models form the basis for advanced three-dimensional radiative transfer simulations, including ray-tracing methods based on Monte Carlo techniques.

A key application of canopy radiative transfer models is retrieval of crop biophysical parameters from remote sensing data. By constructing forward models to simulate reflectance and generating a look-up table (LUT), essential parameters such as LAI and chlorophyll content (Cab) can be estimated through spectral matching with field observations. Alternatively, inversion can be achieved via fitting or optimization algorithms, such as least squares, genetic algorithms, or Bayesian inference. Due to their strong physical interpretability, these models enhance the generalizability and cross-site applicability of remote sensing monitoring frameworks. For instance, in agricultural water stress assessments, radiative transfer models facilitate crop water status evaluation by retrieving key canopy attributes such as Cab, LAI, and canopy chlorophyll content (CCC). Yang et al. [113] developed a water status monitoring framework by integrating UAV-based multispectral imagery with the PROSAIL model. The retrieved parameters—Cab, LAI, and CCC—showed strong correlations with stomatal conductance (Gs). Furthermore, incorporating meteorological factors into the model improved prediction accuracy, demonstrating the robustness and utility of the PROSAIL model when coupled with weather variables for Gs estimation across growth stages. Pasqualotto et al. [114] proposed two indices—the Water Absorption Area Index (WAAI) and the Derivative Water Index (DWI)—by combining PROSAIL-simulated spectra with hyperspectral imagery. Under heterogeneous crop conditions, these indices substantially outperformed traditional ones in estimating canopy water content, achieving R^2^ values of 0.80 and 0.70, respectively. These results highlight the potential of PROSAIL-based indices for scalable remote sensing applications in crop water status monitoring.

Similarly, canopy radiative transfer models have demonstrated strong generalizability and physical advantages in nutrient monitoring. Tripathi et al. [115] employed the PROSAIL model to estimate the LAI and average leaf angle (ALA) of mustard crops by constructing a LUT that linked simulated directional reflectance with canopy structural parameters. Their results confirmed the effectiveness of radiative transfer models in retrieving crop physiological traits. Li et al. [116] proposed the N-PROSAIL model, which integrates a nitrogen-specific PROSPECT model with the SAIL model to enable accurate retrieval of winter wheat nitrogen status at both the leaf and canopy levels (i.e., LNC and CND), outperforming conventional VI-based approaches. Du et al. [107] further combined PROSAIL-simulated spectra with a deep neural network to develop a joint estimation model for LCC and LAI. By incorporating an incremental learning strategy, the model enabled cross-crop nitrogen diagnosis for soybean, rapeseed, and wheat, demonstrating the feasibility of integrating DL with physically based models. Li et al. [117] recently proposed the PROSAIL-NAM framework, which integrates the PROSAIL-PRO with a nitrogen allocation model. In this framework, CNC is divided into photosynthetic and non-photosynthetic components, driven by CCC and CDM, respectively. This approach achieved accuracy CNC estimation across different ecosystems and remote sensing platforms (RMSE = 0.49–2.25 g/m^2^), offering methodological support for crop nitrogen monitoring at regional to global scales.

#### 3.3.2. Microwave Radiative Transfer Models

In contrast to optical remote sensing, which depends on solar radiation as the primary energy source, microwave remote sensing acquires data through active emission (e.g., SAR) or passive sensing of land surface emissions (e.g., microwave radiometers). This capability enables continuous, all-weather monitoring and provides advantages for observing crop–soil systems under cloudy or rainy conditions. Microwave radiative transfer models simulate the propagation, scattering, absorption, and attenuation of microwave signals through vegetation and soil. These models facilitate the retrieval of key biophysical parameters, particularly those sensitive to microwave signals through soil and vegetation.

The interaction between microwave signals and the land surface is influenced by factors such as soil volumetric water content, surface roughness, vegetation water content, structural density, polarization, and incidence angle. Microwave radiative transfer models describe these interactions using electromagnetic propagation and scattering theories, such as Maxwell’s equations and the radiative transfer equation. Their primary aim is to establish quantitative linkages between microwave observations and biophysical surface parameters. Based on the simulation targets and underlying mechanisms, these models are categorized into soil scattering models and coupled vegetation–soil models.

The Integral Equation Model (IEM) is one of the most widely adopted physically based models for simulating microwave backscatter over bare or sparsely vegetated surfaces [118]. It conceptualizes the land surface as a rough dielectric interface and calculates backscatter under varying polarizations and incidence angles by incorporating surface conductivity, geometric descriptors, and both volume and surface scattering. IEM has been extensively applied in multi-frequency (e.g., C-, L-, and X-band) and multi-polarization SAR analyses, serving as a key approach for surface soil moisture retrieval. However, accurate implementation necessitates detailed measurements of surface roughness parameters—such as root mean square (RMS) height and correlation length—rendering it experimentally demanding. To enhance practicality, many studies adopt simplified assumptions or calibrate the model with in situ observations [119,120].

In vegetated environments, microwave signals are influenced by both soil backscatter and vegetation absorption, scattering, and attenuation. Under these conditions, soil scattering models alone fail to fully capture the composite microwave response. To address this limitation, coupled models have been developed that incorporate both vegetation and soil contributions. The Water Cloud Model (WCM), a widely used empirical approach, treats vegetation as a homogeneous water cloud and characterizes its microwave interactions using exponential functions [121]. WCM expresses total backscatter as the sum of vegetation and soil components and is commonly applied in low-relief, uniformly vegetated areas such as croplands and grasslands. Its main advantages include structural simplicity and minimal parameterization, making it well suited for integration with ML–based inversion frameworks. In contrast, the Michigan Microwave Canopy Scattering Model (MIMICS) is a physically based model that simulates multiple scattering within crop canopies and the underlying soil [122]. It accounts for volume scattering, canopy geometry, and dielectric variability. Unlike the empirical WCM, MIMICS supports multilayer canopy representation, making it suitable for complex structures and medium-to-high vegetation densities. However, it requires more detailed inputs and higher computational cost.

Microwave radiative transfer models are extensively employed to retrieve key parameters of crop water status, such as SMC, vegetation water content (VWC), and aboveground biomass (AGB). For instance, Romshoo et al. [123] conducted a typical C-band microwave remote sensing experiment using a scatterometer to collect radar backscatter signals from vegetated surfaces under multi-temporal conditions. By integrating IEM, volume scattering, and empirical models, the study evaluated the effects of soil moisture and vegetation variables on radar backscatter. The results confirmed the sensitivity of microwave signals to both soil and vegetation water content, particularly under different biomass conditions. Zhang et al. [124] developed an SMC retrieval framework using multi-frequency SAR data. By integrating an improved CIEM with a lookup table optimization algorithm, the framework avoided predefined surface roughness parameters—a common limitation in traditional models. The method demonstrated strong estimation accuracy across multiple agricultural bare-soil sites, significantly improving the robustness and practicality of microwave-based physical models. Yahia et al. [125] further fused SAR, multispectral, and TIR data to construct a neural network-based framework for SMC estimation. By implementing a joint weighting strategy at both feature and decision levels, the framework incorporated EA-IEM outputs with PDI and TVDI indices. It was validated across three agricultural sites with distinct soil characteristics in the UK and Algeria, achieving high accuracy and generalizability. These findings highlight the potential of multi-source remote sensing fusion for improving SMC retrieval.

#### 3.3.3. Energy Balance Models

Energy balance models are physically based approaches that quantify the partitioning of surface energy and water fluxes by simulating solar radiation absorption, longwave radiation exchange, and heat transfer processes within soil and vegetation. These models are based on the principle that incoming solar radiation at the land surface is either converted into latent heat through evaporation and transpiration (evapotranspiration, ET), or dissipated as sensible heat. Based on the attribution of energy fluxes to surface components, energy balance models are classified into single-source and dual-source types.

Single-source models treat the land surface as a homogeneous medium, without distinguishing heat exchange between soil and vegetation. These models are most applicable in areas with low vegetation cover or spatially uniform soil–vegetation energy interactions. The Surface Energy Balance Algorithm for Land (SEBAL) is a representative single-source model that estimates surface energy fluxes based on remote sensing inputs, such as surface albedo, radiative fluxes, and land surface temperature [126]. The Mapping Evapotranspiration at High Resolution with Internalized Calibration (METRIC) model extends SEBAL by incorporating high-resolution remote sensing data and introducing an internal calibration mechanism for automatic parameter adjustment [127].

Dual-source energy balance models distinguish between energy contributions of soil and vegetation, enabling the separate estimation of soil evaporation and plant transpiration. These models are suitable for areas with high vegetation cover, such as crop fields or forests, where soil and vegetation contribute unequally to evapotranspiration. The Two-Source Energy Balance (TSEB) model is a representative example. It independently calculates sensible and latent heat fluxes from soil and vegetation, allowing for more accurate simulation of surface energy partitioning in densely vegetated areas [128].

The key outputs of energy balance models are ET and crop water use efficiency (WUE), both of which are vital indicators for assessing crop growth and water consumption. With advances in UAV-based TIR remote sensing, integrating UAV-derived LST data into the TSEB model facilitates high-precision ET estimation. Research has shown that the TSEB model, along with its improved variant DTD, which incorporates LST error correction, can accurately simulate ET under both clear and cloudy conditions in barley fields. The results showed strong consistency with eddy covariance observations, confirming the feasibility and accuracy of applying UAV-based thermal imagery in energy balance modeling [129]. UAV-based multispectral remote sensing, when integrated with energy balance models, has also demonstrated strong capabilities in ET monitoring. For instance, multispectral UAV imagery combined with the SEBAL was used to estimate ET in a pistachio orchard in an arid region, generating high-resolution ET and crop coefficient (Kc) maps at 10 cm spatial resolution. The Kc maps, derived using NDVI and the FAO-56 guidelines, enabled precise identification of drought-affected trees, thereby providing technical support for precision irrigation and drought response strategies [130].

Moreover, UAV-based remote sensing enables high-throughput and non-destructive assessment of WUE. Na et al. [131] integrated the SEBAL model, Kc, and a soil water balance equation to estimate daily ET through the growth period of winter wheat. They also used UAV-derived multispectral data combined with the RF algorithm to retrieve aboveground biomass, enabling quantification of WUE under different irrigation regimes across multiple wheat cultivars. The results indicated that biomass-based WUE at the flowering stage was highly correlated with final yield WUE, providing an effective indicator for selecting water-saving cultivars and demonstrating the potential of UAVs in precision breeding and agricultural water-saving management.

### 3.4. Hybrid Models

With advancement in remote sensing technologies and the diversification of data acquisition methods, the limitations of purely physically based models in accuracy and applicability have become increasingly evident. This is especially evident under complex environmental conditions, where physical models struggle to account for the intricate interactions among influencing factors. In contrast, ML algorithms excel at processing large-scale, high-dimensional datasets but lack physical interpretability. As a result, integrating physical models with data-driven approaches—such as ML and DL—to form hybrid models has emerged as a promising strategy for improving the accuracy and generalizability of remote sensing-based crop monitoring.

Hybrid models preserve the physical interpretability of process-based models while incorporating the adaptive capabilities of ML algorithms, thereby achieving high predictive accuracy and robustness under varying environmental conditions. One common approach embeds ML algorithms within physical models, using outputs of physical simulations as inputs for data-driven inversion or parameter optimization. Alternatively, ML can train and calibrate key parameters within physical models, enhancing adaptability to complex and heterogeneous field conditions. For example, Impollonia et al. [132] compared multiple PROSAIL inversion strategies and proposed a hybrid method that combines physical model simulations with ML regression. This approach improved the estimation accuracy of LCC and LAI in industrial hemp under different nitrogen treatments, confirming the values of physically simulated data in enhancing the performance of ML models during the training phase. Ling et al. [133] addressed the spectral discrepancy between simulated and measured data in traditional hybrid models by proposing an iterative hybrid inversion method. The approach employs a BPNN for inversion, then iteratively optimizes PROSAIL parameters using the inversion results, generating training samples that better resemble real remote sensing imagery. This iterative process progressively enhances model accuracy and improves the stability and robustness of LAI estimation for winter wheat. Bhadra et al. [134] incorporated PROSAIL-simulated data as prior knowledge into a neural network and applied a transfer learning strategy to enhance the model’s generalization capability on real UAV-based hyperspectral imagery. This architecture, which combines DL with physically based modeling, leverages the physical interpretability of PROSAIL and improves the estimation accuracy of LCC and ALA under complex agricultural field conditions through a 1D-CNN.

### 3.5. Comparative Evaluation of Modeling Approaches

Although diverse modeling approaches—including VI-based, data-driven, physically based, and hybrid models—have been applied to UAV remote sensing of crop water and nutrient status, their comparative performance varies under different agricultural conditions. VI-based methods are computationally simple and interpretable but often suffer from spectral saturation and limited robustness in heterogeneous fields. Data-driven models achieve high predictive accuracy and flexibility, yet their effectiveness strongly depends on large, high-quality training datasets, and they often lack interpretability. Physically based models exhibit stronger generalizability across regions and crops because they are grounded in mechanistic principles, but they usually demand extensive parameterization and are computationally intensive. Hybrid models represent a promising pathway by combining the interpretability of physical models with the adaptability of AI, thereby improving robustness and transferability; however, their operational applications are still at an early stage. A comparative summary of these modeling approaches is presented in Table 4.

## 4. Key Factors Affecting UAV-Based Remote Sensing of Crop Water and Nutrient Status

With the widespread application of UAV-based remote sensing in agricultural water and nutrient monitoring, data acquisition accuracy has improved significantly. However, practical implementation still faces many challenges. The key factors affecting monitoring performance include crop canopy coverage and growth stages, spatial resolution and scale compatibility, environmental disturbances, and data processing and model generalization capability.

### 4.1. Influence of Canopy Coverage and Growth Stage

Canopy coverage and growth stages jointly determine the structural and physiological characteristics of crop canopies and are key factors affecting the accuracy of UAV-based remote sensing for monitoring water and nutrient status. As crops develop from emergence to maturity, canopy structures become dense, accompanied by significant changes in spectral and radiative properties. These dynamics directly influence the remote sensing response and the accuracy of inversion models.

At early growth stages with low vegetation coverage, exposed soil dominates the field surface and introduces background interference to remote sensing signals. For example, in optical remote sensing, even after image segmentation removes most soil pixels, mixed pixels remain at canopy–soil boundaries, weakening the sensitivity of VIs such as NDVI to canopy variation [135]. As vegetation coverage increases and the canopy closes, soil interference is reduced. However, overlapping of multiple leaf layers can lead to optical saturation. Specifically, VIs such as NDVI and EVI become insensitive to further increases in LAI once it exceeds a threshold (typically around 4), resulting in a plateau effect [136,137], which compromises the accuracy of LAI and chlorophyll content retrieval [138]. For example, in fields with high vegetation coverage, spectral saturation effects can cause large estimation errors, with the normalized root mean square error (NRMSE) of potato aboveground biomass exceeding 20% [139].

More importantly, transitions between crop growth stages alter canopy structure and induce temporal shifts in water and nutrient responses. During the vegetative stage, nitrogen demand dominates, and reflectance in the red-edge (700–750 nm) and green bands is highly sensitive to nitrogen variation [140]. In contrast, during the reproductive stage, water becomes the primary limiting factor, and TIR signals as well as NIR reflectance show stronger responses [141]. These stage-dependent spectral sensitivities challenge the development of universally applicable models for water and nutrient inversion.

### 4.2. The Impact of Scale Effects and Spatial Resolution

The monitoring complexity introduced by variations in vegetation coverage and growth stages is further compounded by the inherent characteristics of UAV-based remote sensing. While UAVs offer centimeter-level spatial resolution for fine-scale agricultural monitoring, this precision also leads to a “scale mismatch” problem when applied to field-level management practices. Specifically, the spatial scale of data acquisition often does not align with the scale of agricultural decision-making, resulting in scale effects. These effects compromise the accuracy of parameter inversion and limit the generalization and scalability of models for regional applications.

The mixed pixel problem remains prevalent even under high-resolution conditions. Although UAV-based imagery can achieve spatial resolutions of 1–10 cm, a single pixel may still contain multiple ground components, such as vegetation, bare soil, shadows, and water bodies, resulting in spectral mixing and distortion of water or nutrient indices [142]. In the optical domain, reflectance differences among surface features are substantial. In fields with low canopy cover or wide inter-row spacing, the combined reflectance of soil and vegetation can easily lead to misestimation of water-related indices [143]. Spatial heterogeneity is especially prominent at the scale of management units (e.g., plots or fields), where crop water status or nutrient availability often shows patchy, non-uniform distribution. Such local stress may result from factors like groundwater variation or uneven fertilizer application [144]. At low spatial resolution (e.g., >1 m), pixel averaging may obscure localized stress signals, and result in missed detections. Although high-resolution imagery enables detailed observation, the challenge remains how to effectively aggregate such fine-scale information into actionable insights at the management scale.

### 4.3. Effects of Illumination, Meteorology, and Other Environmental Factors

In addition to crop characteristics and observational scale, variations in illumination and atmosphere are major external factors contributing to data uncertainty and inversion errors in field-scale remote sensing. UAV-based remote sensing primarily relies on passive optical and TIR sensors to capture surface reflectance and thermal radiation, making its observations highly susceptible to interference from factors such as solar angle, wind, and atmospheric humidity [145,146]. These environmental variables vary across temporal and meteorological conditions, and if left uncorrected, can lead to systematic misinterpretation in crop water and nutrient monitoring. Specifically, changes in solar elevation directly influence the amount of incoming radiation received by optical and thermal sensors. During early morning and late afternoon, the low solar angle increases shadow coverage between surface features, particularly in ridged farmland, which causes diurnal fluctuations in VIs and compromises the stability of physiological assessments of crops [147].

Similarly, variations in wind speed, air temperature, and humidity directly affect TIR remote sensing. TIR sensors are widely used to monitor canopy temperature, serving as a critical tool for estimating evapotranspiration (ET) and diagnosing water stress. When wind speed exceeds 2 m/s, increased canopy turbulence and air mixing reduce the sensitivity of thermal imagery to water stress conditions [148]. Therefore, concurrent monitoring and correction of environmental parameters are essential for ensuring the reliability of remote sensing assessments.

## 5. Challenges and Future Prospects for UAV-Based Monitoring of Crop Water and Nutrient Status

With the advances of agricultural remote sensing systems, UAV platforms and AI algorithms have been widely applied to monitor crop water and nutrient status. However, several technical and application bottlenecks persist in practical implementation. These challenges span from sensor performance at the hardware level to the efficiency of data processing workflows and the adaptability and interpretability of monitoring models. To better support precision field management, it is imperative to systematically identify and address the following limitations.

### 5.1. Current Challenges

(1) **Delayed development of proximal remote sensing sensors and insufficient specialization and adaptability.** At present, spectral sensors used for crop water and nutrient monitoring are still dominated by general-purpose multispectral or TIR devices, lacking wavelength configurations and structural designs optimized for agricultural scenarios. For example, high-resolution sensors tailored to key spectral bands sensitive to crop water and nutrient status have not yet been developed, making it difficult to detect subtle changes in crop physiological conditions. In addition, under complex field conditions such as high temperature, humidity, and wind, current sensors often suffer from limited stability and adaptability, which restricts both data quality and monitoring frequency.
(2) **Lengthy data processing chains with limited real-time performance and intelligence.** Although UAV-based remote sensing platforms offer high-resolution observation capabilities, the massive volume and diversity of the acquired imagery still require complex and time-consuming post-processing workflows. These steps include image mosaicking, radiometric correction, and parameter inversion, which are often labor-intensive and heavily dependent on manual intervention. These workflows hinder timely analysis, reduce data utilization efficiency, and fail to meet the rapid response demands of agricultural decision-making or support high-frequency, dynamic monitoring tasks.
(3) **Insufficient depth in multi-source data fusion and underutilization of spatial information.** Current research efforts are largely focused on processing data from a single platform, with limited integration of remote sensing information across multiple platforms—including satellites, UAVs, and ground-based sensors—and modalities such as optical, TIR, and radar. Particularly, systematic fusion methods are lacking in areas such as scale transformation, temporal gap-filling, and canopy structural reconstruction. This limits the ability to capture in-field variability and support regional-scale decision-making, constraining the spatial adaptability of precision agriculture.
(4) **Significant environmental interference in remote sensing inversion leads to high data uncertainty.** UAV-based remote sensing primarily relies on passive sensors to capture surface reflectance, making it highly susceptible to solar angle, wind speed, atmospheric humidity, and cloud cover. During the early growth stages, strong soil background signals may obscure crop spectral features, while in later stages, dense canopy overlap can result in spectral saturation. Moreover, shadow occlusion and terrain variation further challenge radiometric consistency. Without correction, these factors can compromise the stability and reliability of model outputs.
(5) **Limited model generalization, cross-regional adaptability, and interpretability.** Most existing models rely heavily on locally trained datasets, making them difficult to generalize across different crops, regions, seasons, and management practices. In complex agricultural environments, these models are prone to overfitting and transfer failures. Although DL-based “black-box” models often achieve high accuracy, they lack explicit physiological or physical interpretability, undermining their credibility in intelligent field diagnostics. This limitation hinders their practical deployment and scalability in real-world agricultural management.
(6) **Practical barriers in real-world adoption.** Despite the rapid development of UAV-based remote sensing technologies, their widespread adoption in agricultural practice faces several practical barriers. High initial investment costs for UAV platforms and sensors, as well as the need for trained personnel to operate and maintain these systems, often limit their accessibility for smallholder farmers. In addition, regulatory restrictions—such as flight permits, operational safety requirements, and data privacy concerns—pose institutional challenges, especially in regions with evolving UAV policies. These factors hinder the scalability and routine use of UAV-based diagnostics, emphasizing the need for cost-effective solutions, simplified user interfaces, and policy support frameworks to facilitate broader implementation.

### 5.2. Future Prospects

(1) **Development of application-specific sensors and edge-intelligent devices agricultural scenarios.** To address the limitations of general-purpose sensors in crop monitoring, future efforts will focus on developing sensor modules designed to capture crop-sensitive spectral bands related to water and nutrient status. These sensors will be optimized for lightweight design, low power consumption, and enhanced resistance to field interference. Moreover, smart terminals with edge computing modules—such as UAVs or in-field sensor nodes—will enable real-time data processing, including image stitching, VI computation, and key region extraction during flight. Only essential information will be transmitted to the cloud, thereby reducing bandwidth demand and latency. This approach ensures stable, high-frequency sensing data to support in-field diagnosis of crop water and nutrient status.
(2) **Establishing an edge–cloud collaborative architecture to enhance processing efficiency and intelligence.** Leveraging 5G communication and AI algorithms, a new dual-layer architecture can be established for UAV-based remote sensing that integrates data acquisition, transmission, and processing. This architecture consists of edge-level preprocessing and cloud-based deep analysis. Onboard UAV systems will handle basic tasks such as noise reduction, VI computation, and target segmentation, while high-complexity operations—such as radiometric correction, inversion modeling, and large-scale data analysis—are performed in the cloud. This division improves processing efficiency, enables near real-time diagnosis, and promotes the transformation of UAV-based remote sensing from a “data acquisition tool” to an “intelligent decision-making system.”
(3) **Multi-scale collaborative remote sensing and 3D data fusion to enhance spatial awareness and decision support.** Integrating UAVs, satellites, and ground-based platforms enables unified crop monitoring across centimeter- to kilometer-scale resolutions. Combining LiDAR and multispectral imagery, high-precision 3D canopy models can be generated, allowing joint feature extraction of crop height, canopy structure, and spectral responses. Based on these models, AI models can automatically detect spatially heterogeneous regions, such as zones of water stress or uneven fertilization. These outputs support precision fertilization and site-specific irrigation, improving the efficiency and operability of precision agriculture.
(4) **Developing dynamic correction mechanisms to improve environmental adaptability and reduce remote sensing uncertainty.** To address the common environmental interferences in remote sensing inversion, adaptive models and correction frameworks should be developed, including coverage-adaptive models, physiology–spectrum coupled models, and real-time environmental correction systems. For instance, in the early growth stage, soil reflectance modeling can reduce spectral contamination, while in the grain-filling stage, multi-angle imaging can alleviate saturation effects. Additionally, integrating real-time meteorological variables—such as wind speed, humidity, and solar radiation—can guide radiometric correction algorithms to perform shadow compensation, angle normalization, and wind–temperature coupling adjustment, improving data consistency and reliability across time and space.
(5) **Integrating physical mechanisms with AI for interpretable models to improve generalization and robustness.** To overcome limitations in model transferability and interpretability, a “pretraining–fine-tuning” paradigm can be adopted. Large-scale, multi-crop datasets can build generalized base models, which are then rapidly adapted to specific regions or crops using small local samples. Furthermore, integrating physical priors with neural networks—such as embedding radiative transfer models (e.g., PROSAIL) into the architecture—enables the incorporation of physical constraints during training. This hybrid approach enhances model stability, interpretability, and data efficiency, facilitating a transition from opaque “black-box” systems to transparent, knowledge- and data-driven solutions in agricultural remote sensing.
(6) **Promoting practical deployment through cost-effective design, policy support, and user-friendly tools.** To overcome real-world barriers, future research and development should prioritize cost-effective UAV systems and simplified operational workflows tailored for agricultural end-users. Developing modular, low-cost UAV platforms with plug-and-play sensors can reduce entry barriers for small and medium-sized farms. In parallel, intuitive software interfaces and semi-automated workflows will lower the technical threshold for non-expert users. Moreover, establishing supportive regulatory frameworks and providing training programs or service outsourcing models will facilitate broader and safer UAV adoption in agricultural practice, bridging the gap between research innovations and field-level implementation.

## Figures and Tables

**Table 1:** UAV platform types and their technical parameters for agricultural monitoring.

Rotor Type	Endurance Time	Maximum Altitude	Equipment Cost	Maintenance Complexity	Typical Platform
Multirotor	<1 h	Within a few hundred meters	Relatively low	Relatively simple	DJI M300
Fixed-wing	Several hours	Several kilometers	Moderate	Moderate	HC-141
Hybrid-wing	Several hours	Several kilometers	Relatively high	Relatively complex	CW-100

**Table 2:** Classification and technical parameters of remote sensing sensor types.

Sensor Type	Spectral Range	Typical Payload	Applications
Optical remote sensing	0.4–1.1 μm (Visible/NIR)	Micasense RedEdge-P	Vegetation index-based assessment of nitrogen status; estimation of LAI
Hyperspectral sensor	0.4–2.5 μm (Hundreds of narrow bands)	Cubert ULTRIS 5	Inversion of soil organic matter; early detection of crop pests and diseases; nutrient deficiency diagnosis
Thermal infrared sensor	8–14 μm	FLIR Vue Pro	Monitoring canopy temperature for water stress; estimation of surface water content via thermal inertia
Microwave remote sensing	0.1 cm–1 m (C/X/L bands)	FSAR miniSAR	Soil moisture inversion; crop biomass estimation; flood and waterlogging monitoring

**Table 3:** Common vegetation indices and their formulas.

Vegetable Index	Formula	Reference
Drought Stress Index (DSI)	$DSI=\frac{S_{1}}{N}$	[56]
Difference Vegetation Index (DVI)	DVI=N−R	[57]
Enhanced Vegetation Index (EVI)	$EVI=\frac{g*(N-R)}{\left(N+C_{1}R-C_{2}B+L\right)}$	[58]
Excess Green Index (ExG)	EXG=2∗G−R−B	[59]
Green Normalized Difference Vegetation Index (GNDVI)	$GNDVI=\frac{\left(N-G\right)}{\left(N+G\right)}$	[60]
Normalized Difference Moisture Index (NDMI)	$NDMI=\frac{\left(N-S_{1}\right)}{\left(N+S_{1}\right)}$	[61]
Normalized Difference Vegetation Index (NDVI)	$NDVI=\frac{\left(N-R\right)}{\left(N+R\right)}$	[62]
Hyperspectral Near-Infrared Reflectance of Vegetation (NIRvH2)	NIRvH2=N−R−k(λN−λR)	[63]
Optimized Soil-Adjusted Vegetation Index (OSAVI)	$OSAVI=\frac{\left(N-R\right)}{\left(N+R+0.16\right)}$	[64]
Ratio Vegetation Index (RVI)	$RVI=\frac{{RE}_{2}}{R}$	[65]
Soil-Adjusted Vegetation Index (SAVI)	$SAVI=\frac{\left(1.0+L)(N-R\right)}{\left(N+R+L\right)}$	[66]
Transformed Difference Vegetation Index (TDVI)	$TDVI=\frac{1.5(N-R)}{{\left(N^{2}+R+0.5\right)}^{0.5}}$	[67]
Transformed Vegetation Index (TVI)	$TVI={\left(\frac{\left(N-R\right)}{\left(N+R\right)}+0.5\right)}^{0.5}$	[68]

Note: N = near-infrared reflectance; R = red reflectance; G = green reflectance; *B* = blue reflectance; ${RE}_{2}$ = red-edge reflectance; $S_{1}$ = shortwave infrared reflectance; λN and λR = central wavelengths of NIR and red bands; k = correction factor; L = soil adjustment factor; g = gain factor; $C_{1}$ and $C_{2}$ = atmospheric correction coefficients.

**Table 4:** Comparative summary of modeling approaches for UAV-based monitoring of crop water and nutrient status.

Modeling Approach	Accuracy	Scalability	Data Requirements	Robustness	Advantages
VI-based methods	Moderate; sensitive to canopy coverage	High; easy to implement	Low (few spectral bands)	Limited under heterogeneous or stressed conditions	Simple, interpretable, cost-effective
Data-driven models	High with sufficient training data	Moderate; limited cross-region transfer	High (large, labeled datasets required)	Variable; prone to overfitting	Nonlinear modeling capacity, flexible
Physically based models	Moderate-high; depends on parameterization	High; applicable across crops and regions	Medium-high (field + meteorological inputs)	Strong generalization across conditions	Mechanistic, interpretable
Hybrid models	High; balance of accuracy and interpretability	Promising, but not fully validated	Medium-high (multi-source data required)	Strong; potential for transferability	Combine physical priors with AI adaptability
